# Experimental and Numerical Analysis of Stitched Composite Laminates Subjected to Low-Velocity Edge-on Impact and Compression after Edge-on Impact

**DOI:** 10.3390/polym15112484

**Published:** 2023-05-27

**Authors:** Bangxiong Liu, Jiamei Lai, Hesheng Liu, Zhichao Huang, Bin Liu, Ze Peng, Wei Zhang

**Affiliations:** 1 Polymer Processing Research Laboratory, School of Advanced Manufacturing, Nanchang University, Nanchang 330031, China; liubangxiong0798@126.com (B.L.); liubinlpp@163.com (B.L.); pz18271660785@163.com (Z.P.); 2 School of Mechanical and Electronic Engineering, Jingdezhen University, Jingdezhen 333400, China; 3 School of Mechatronics and Vehicle Engineering, East China Jiao Tong University, Nanchang 330013, China; hzcosu@163.com (Z.H.); weizhang@email.ncu.edu.cn (W.Z.)

**Keywords:** stitched composite laminate, edge-on impact, damage failure mechanism, numerical simulation, residual compression strength

## Abstract

Composite laminates are susceptible to impact events during use and maintenance, affecting their safety performance. Edge-on impact is a more significant threat to laminates than central impact. In this work, the edge-on impact damage mechanism and residual strength in compression were investigated using experimental and simulation methods by considering variations in impact energy, stitching, and stitching density. The damage to the composite laminate after edge-on impact was detected in the test by visual inspection, electron microscopic observation, and X-ray computed tomography techniques. The fiber and matrix damage were determined according to the Hashin stress criterion, while the cohesive element was used to simulate the interlaminar damage. An improved Camanho nonlinear stiffness discount was proposed to describe the stiffness degradation of the material. The numerical prediction results matched well with the experimental values. The findings show that the stitching technique could improve the damage tolerance and residual strength of the laminate. It can also effectively inhibit crack expansion, and the effect increases with increasing suture density.

## 1. Introduction

Carbon fiber reinforced polymer (CFRP) is widely used in the aviation, aerospace, and wind power industries because of its high specific strength and stiffness [1,2,3,4]. These composites may be subjected to outside impacts such as bird collisions and hail impacts during work, which are called high-velocity impacts. As the area of damage caused by the high-velocity impact is relatively apparent, it can often be detected [5,6]. However, the potential threat often comes from invisible damage, such as tools falling during overhaul or gravel splashing up during takeoff or descent. These minor damages may not be observed or noticeable and are often overlooked by aircraft maintainers. Nevertheless, delamination and matrix cracking may have occurred within the material, which can significantly reduce the strength of the structure [7,8,9,10].

Due to the above reasons, improving the damage tolerance of carbon fiber composites has attracted much interest from scholars [11,12,13,14,15,16,17,18,19,20,21,22,23], who have improved the impact damage tolerance of the composites through structural design and particle modification. Sonnenfeld et al. [11] proposed a way to insert thermoplastic materials into thermoset laminates, which can dissipate impact damage and enhance the impact behavior of composites. Ravindran et al. [12] and Fenner et al. [13] effectively increased the damage tolerance by using the particle-reinforced method. The stitching [14,15,16,17,18,19,20] and Z-pin [21] processes have been adopted by researchers to improve the weak interlaminar behavior of composites. The delamination resistance of the composite was improved by inserting stitching or a Z-pin into the laminate, and this method was used to effectively inhibit the extension of delamination cracks. Lin et al. [24] and Wang [25] investigated the effect of a negative Poisson’s ratio on the low-velocity impact of carbon fiber composite laminates through numerical simulation. The results showed that the negative Poisson’s ratio structure helps reduce the tensile damage of the fibers and matrix and the delamination damage area. Falaschetti et al. [26] studied the near-edge and central impacts of laminates in a hydrothermal environment. They showed that the effect of hydrothermal aging on the compressive strength of laminates was greater than the effect of invisible damage on the compressive strength of laminates. Biagini et al. [27] proposed an information identification mechanism for compression after impact (CAI) damage based on the b-value of the acoustic emission technique. The study results showed that the accumulation of unstable damage occurred at 80% of the failure displacement.

At present, a large amount of the literature investigating the low-velocity impact damage tolerance of composites has focused on the central impacts away from the edges. However, many impact events occurred at the edges during the working process. For example, the steering of an aircraft before takeoff can collide with eaves and maintenance vehicles due to improper handling, causing severe damage [28,29]. Malhotra et al. [30] carried out a study on the edge and near-edge impacts of laminates. They showed that edge impacts have longer fractures and more delamination compared to near-edge impacts, and that these delaminations are fan-like in shape [31]. Ostré et al. [32] noticed that the mechanical response of the edge-on impact is significantly different from that of the center impact, with a “plateau band” of smooth fluctuations in the force-displacement curve. Thorsson et al. [33] studied edge-on impact at 0° and 45° angles. Similar curves were obtained for edge-on impacts at 0° angles, with more noticeable delamination, indicating the presence of heavily concentrated localized damage at the edge-on impact location. Arteiro et al. [34] establish low-velocity impact (LVI) and compression after edge impact (CAEI) models for composites based on the continuum damage mechanics (CDM) theory, which can accurately predict the morphology of damage from edge-on impact. Li et al. [35] proposed a mechanical model considering fracture plane angle within anisotropic materials, which can be matched better with the experimental results. The failure mechanisms of composite materials subjected to edge impact were investigated through experiments and numerical simulations by Liu et al. [36] and Xu et al. [37]. In previous studies, there was little literature about the edge-on impact of stitched composites. The authors experimentally studied the edge-on impact of stitched composites [38], but the failure mechanism has yet to be investigated in further detail. 

This work aims to reveal the failure mechanism of edge-on impact within laminates through experiments and simulations. The effect of the stitching process on the behavior of composite laminates for low-velocity edge-on impact (LVEI) and CAEI was investigated. An original fixture has been used for edge-on impact tests, and standard test fixtures were used for compression tests. The model was established based on CDM theory. The Hashin criterion was used as the initial damage criterion to simulate intralaminar damage. A modified Camanho degradation solution was used for stiffness discounting, and a zero-thickness cohesive element was adopted for delamination damage. The experimental data verified the reliability of the simulation method.

## 2. Experimental Procedure

### 2.1. Specimen Preparation

**Figure 1:**
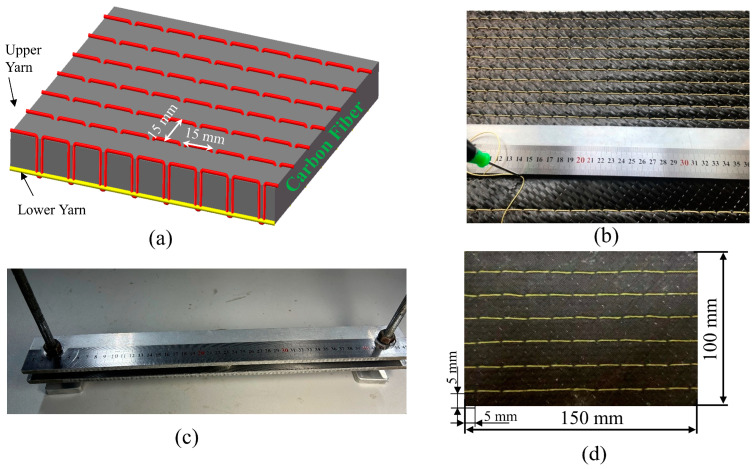
Schematic diagram of the stitching process: (**a**) the modified lock stitching process; (**b**) the process of stitching the prefabricated part; (**c**) the unique tool to assist with manual stitching; (**d**) the impact specimen (150 × 150 mm).

The carbon fibers were CF12-L300 (Zhongfu Shenying Carbon Fiber Co., Ltd., Lianyungang, China), the epoxy was R668 (Nan Ya Plastics Corporation, Taiwan, China), and the curing agent was H3268 (Basf Group, Ludwigshafen, Germany). The stitching yarns were 1500 denier Kevlar-29 (DuPont Group, Wilmington, DE, America). The fiber arrangement was [−45/0/90/45/90]_2s_. A modified locking type was applied to insert the yarn into the preforms, as seen in Figure 1a. We experimented with a manual stitching machine earlier. The stitching effect was not good because the fibers in the fiber fabric were hooked out during the upward movement of the machine needle. For this reason, we designed the unique tool to assist with manual stitching, and the Kevlar yarns were passed through the stacked layers by manual stitching to produce a preform with a 3D fiber structure, as illustrated in Figure 1b,c. The density of the stitched area was divided into 15 × 15 mm and 10 × 10 mm. The preforms were processed into a CFRP laminate by the VARTM forming process method. The epoxy resin and curing agent were mixed in a ratio of 5:1. The configured mixture was held in the resin cup at atmospheric pressure, and the vacuum pump was used to inject the mixture into the prefabricated fiber area inside the vacuum bag. The extra mixture flowed into the resin trap when it was filled. After an atmospheric pressure curing time of 24 h, the CFRP laminate was cut to a standard impact specimen of length 150 mm, width 100 mm, and thickness 6 mm by waterjet, and the fiber volume fraction of the specimen was 52%. The first stitching line from the edge of the impact specimens is 5 mm, as displayed in Figure 1d. The specimens were selected according to the stitching density: unstitched plate (UP), stitched plate with a density of 10 × 10 mm (SP10), and stitched plate with a density of 15 × 15 mm (SP15).

### 2.2. Edge-on Impact Test

**Figure 2:**
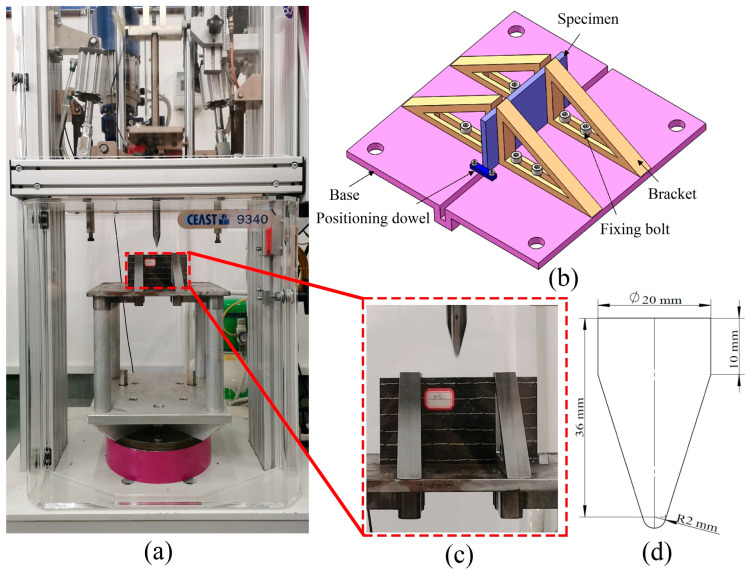
Experimental device for LVEI. (**a**) Instron CEAST 9340 drop-weight impactor; (**b**) CAD model of the edge-on impact device; (**c**) physical model of the edge-on impact device; (**d**) geometric dimensions of the impactor.

An impact machine (Instron CEAST 9340, Instron Corporation, Norwood, MA, America) was used, referring to the ASTM D7136 standard for the edge-on impact tests [39]. As can be seen in Figure 2a, the impact energy of the impactor was determined by the height of the falling impactor. Considering the lack of an experimental fixture for the edge-on impact, we designed an edge-on impact fixture, as presented in Figure 2b, consisting of a steel base, a positioning dowel, some brackets, and bolts. The specimen was placed vertically at the bottom of the base. A side of the specimen was closed to the positioning dowel, the mobile brackets were pushed and clamped to the specimen, and then relevant bolts were locked to ensure that the surface of the specimen was perpendicular to the steel base, thus ensuring the accuracy of the impact point position and satisfying the clamping requirements for a specimen of various thicknesses. A steel wedge-shaped impactor was used, which is shown in Figure 2c. The impactor could completely contact the edge area of the specimen during impact with a mass of 5.5 kg, and the geometry of the impactor is shown in Figure 2d, with a 2 mm radius chamfer at the bottom. The impact energy we used was 5 J, 10 J, and 15 J, with each test repeated three times. The drop weight impact machine calculated the drop height based on the impact energy and converted the gravitational potential energy of the drop weight into kinetic energy. The falling height *h* could be calculated by the formula *E* = *mgh*, where *E* is the impact energy, *m* is the impactor mass of 5.5 kg, and *g* is the acceleration of gravity at 9.81 m/s^2^. The velocity was at its maximum when the impactor was in contact with the laminate, which the equation *E* = 1/2*mv*^2^ could calculate. The values of *h* and *v* at three energies for this test are shown in Table 1. The parameters of displacement, force, and velocity of the impactor were measured and recorded in real-time during the impact by the data acquisition system DAS 64K-SC, which was built into the measuring device.

### 2.3. CAEI Test

**Figure 3:**
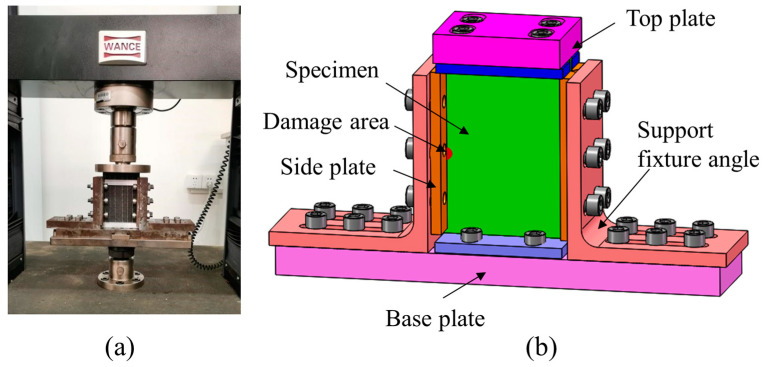
The specimen undergoing compression. (**a**) Physical model of the required support fixture; (**b**) CAD model of the required support fixture.

All the specimens, including non-impact specimens, were compression tested on the ETM105D microcomputer-controlled electronic universal testing machine (Wance Technologies Ltd., Shenzhen, China) to provide the CAI strength. The load scope of this machine is 4–100 kN, and the displacement resolution is 0.025 μm, which can satisfy the experimental requirements. All specimens were compressed according to the ASTM D7137 standard [40], as shown in Figure 3a. A particular fixture was required for CAI tests installed on the universal testing machine. This fixture was mainly used to ensure buckling did not occur during compression loading. The fixture is demonstrated in Figure 3b, and its dimensional accuracy requirements follow ASTM standards. It is assembled with plates and slide plates so that the specimen can be fixed in the middle of the fixture and then clamped with bolts to support the corner plates. The loading rate of the experimental machine is 1.25 mm/min. The tests should be terminated when the load drops to about 70% of the maximum load, indicating that the specimen has failed.

## 3. Experimental Results and Discussion

### 3.1. Edge-on Impact Responses

**Figure 4:**
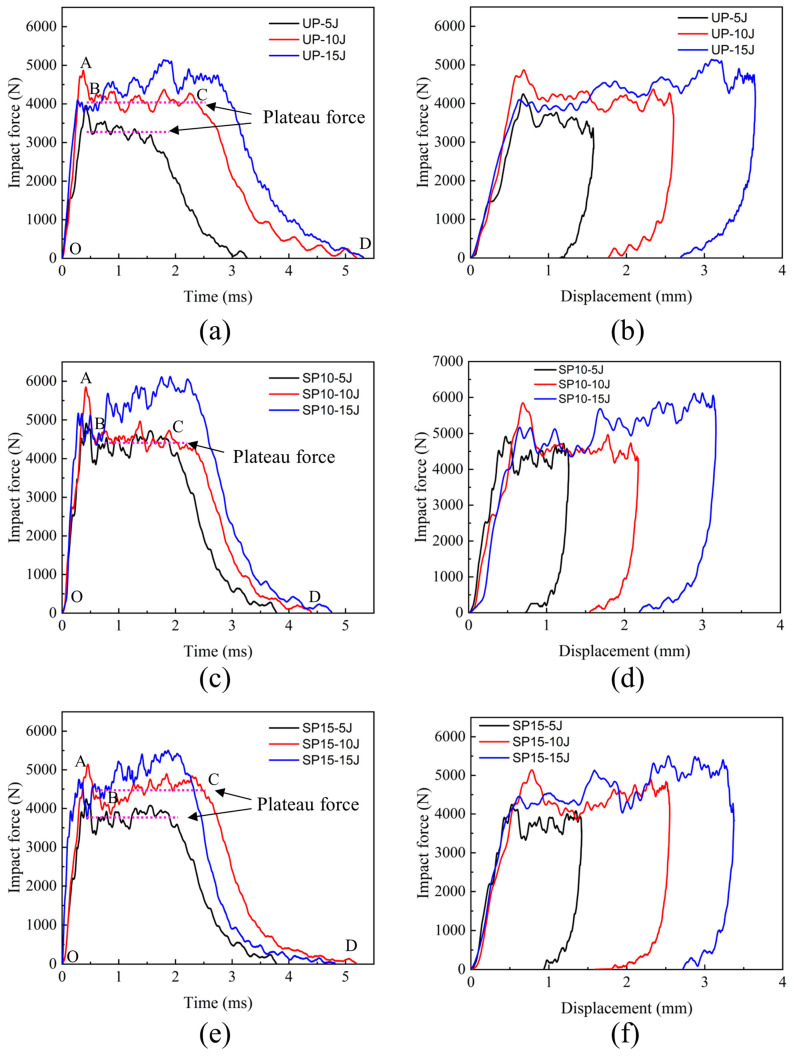
Edge-on impact mechanical responses of three groups: (**a**) force-time curves for group UP; (**b**) force-displacement curves for group UP; (**c**) force-time curves for group SP10; (**d**) force-displacement curves for group SP10; (**e**) force-time curves for group SP15; (**f**) force-displacement curves for group SP15.

The edge-on impact mechanical responses of specimens with stitched and unstitched composite laminates are illustrated in Figure 4. The impact force-time and force-displacement curves for the UP, SP10, and SP15 groups at different energies are indicated, respectively. As seen in Figure 4a,c,e, the stitched and unstitched laminates exhibited similar characteristics at different impact energies, which can be classified into four processes as follows: (1) Phase OA: linear loading phase; (2) Phase AB: peak load abrupt drop; (3) Phase BC: load value stability oscillation; and (4) Phase CD: rebound phase of the impactor. At the beginning of the tup’s contact with the edge of the laminate, the impact force rises in a linear way from zero. Damage accumulates rapidly inside the laminate, and then the impact force increases to a maximum quickly. After arriving at the peak, the load falls off sharply and enters a period of up-and-down oscillation. Finally, the force was gradually unloaded to zero when the punch rebounded. Furthermore, as the impact energy increases to 15 J, the shock plateau phase replaces the peak load sudden drop phase, and a comparatively long displacement segment appears. It may be caused by the accumulation of matrix fragments in the dent created by the impact position in a short time at enormous impact energy, and the internal failure of the laminate begins to propagate downward gradually. It can be seen from Figure 4b,d,f that when the impact force is offloaded to zero, there is a residual displacement that also grows with the increasing impact of energy. This phenomenon demonstrates that the edge of the laminate sustained permanent deformation damage during the impact.

**Figure 5:**
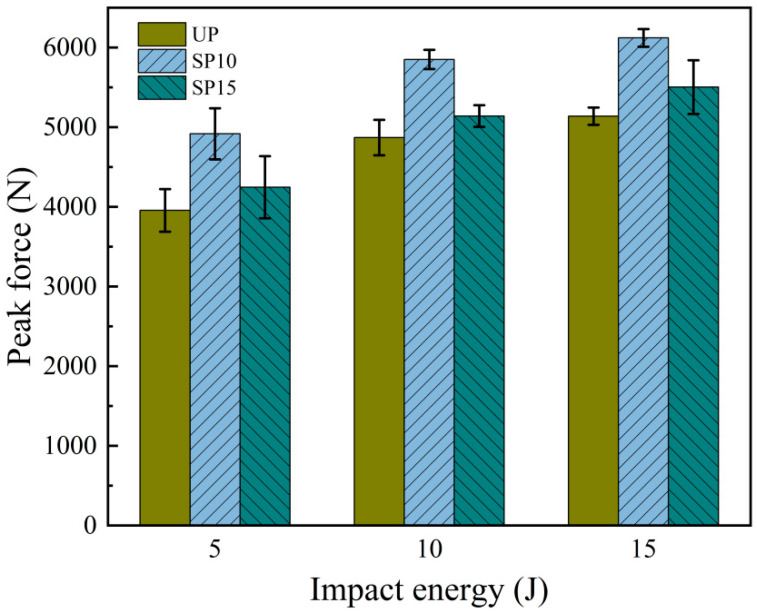
Average peak force values at different edge-on impact energies.

The relationship between the peak impact force and different impact energies for the three groups (group UP, group SP10, and group SP15) is shown in Figure 5. It could be observed from the figure that the average peak impact force of the stitched laminate was always more extensive than the unstitched laminate under the same energy. The mean peak edge-on impact force of the group SP15 increased by 7.37%, 5.56%, and 7.10%, and the group SP10 increased by 24.31%, 20.15%, and 19.12%, respectively, over that of the group UP when the impact energy was 5 J, 10 J, and 15 J. Hence, it was evident that the insertion of stitches was beneficial to enhance the edge-on impact resistance of the laminates. The stitched laminates suffered a higher edge-on impact than the unstitched laminates, and the more densely stitched the laminates were, the more marked the reinforcing effect in these tests.

### 3.2. Surface Damage

**Figure 6:**
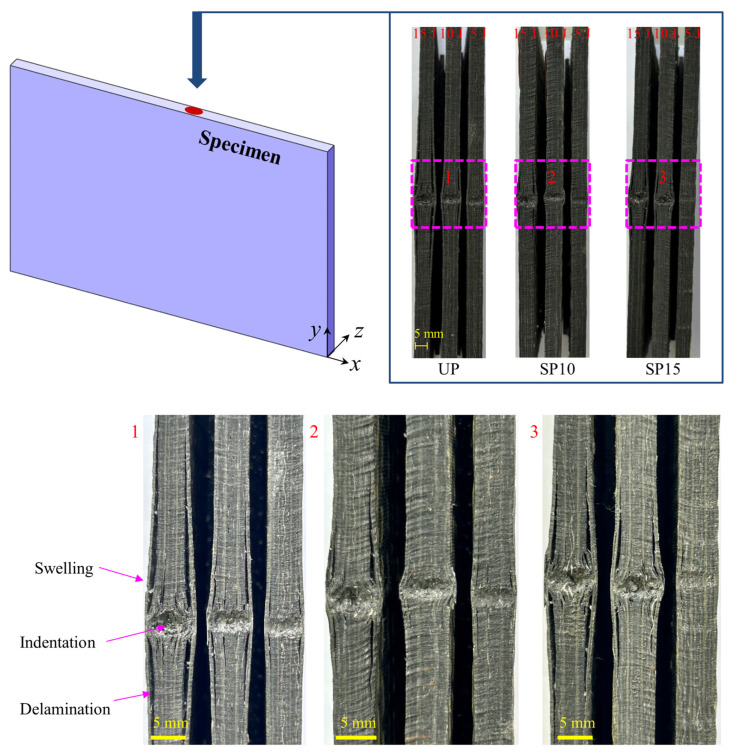
Visual inspection of the surface damage.

To observe the damage to the surface of the composite laminate after an edge-on impact, the surface of the specimens was visually inspected. The typical macroscopic damage morphology at different energies is shown in Figure 6. The apparent damage, such as an impact dent, fiber breakage, and delamination on the composite surface, could be seen by visual observation. The macroscopic damage morphology at the edge of the laminate was relatively similar for different impacting energies and stitching densities. It was observed in the *x-z* plane that, in addition to permanent, irreversible dent damage and out-of-plane swelling at the impact area, there were also delaminated cracks in the *x* direction. As the impact energy increased, the length of the crack on the surface of the specimen increased. The size of the dent becomes larger and deeper, and the expansion becomes more significant than the low-impact energy. The results were similar to those observed in the literature [35], except that this experiment did not show a concentration of damage on one side but was approximately evenly distributed on both sides, in agreement with the results observed in the literature [33]. It was mainly because the impactor used in this test was a wedge tup, and the cylindrical surface of the wedge tup was in contact with the laminate instead of the hemispherical surface. A digital micrometer and microscopy X-ray computed tomography post-processing software were used to measure the length of cracks to evaluate the degree of damage to the laminate. Table 2 gives the crack expansion lengths and the max delamination width of three types of laminates under edge impact, and it can be seen in Table 2 and Figure 6 that the length of damaged cracks increases with the increase in energy. The damage in group UP was the most serious, with the deepest dents and the most extended crack length and expansion at the same impact energy. It indicated that the stitching could improve the stiffness of the laminate and prevent delaminating cracks from spreading. Compared with group SP10 and group SP15, the crack length of group SP10 was shorter than the others. It also has a smaller depth of indentation and less expansion. It shows that the greater the stitching density, the more the extension of delamination can be inhibited.

**Figure 7:**
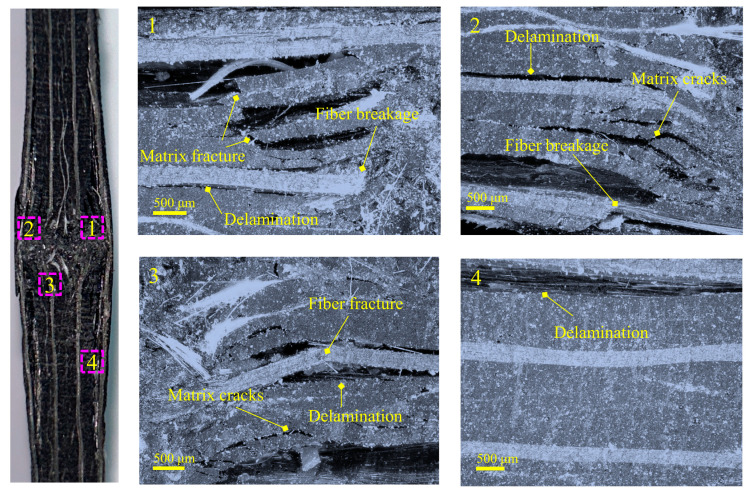
Optical microscopic observation of composite laminate.

Figure 7 demonstrates the morphology of the microscopic damage near the surface around the dent in group SP under an edge-on impact energy of 15 J. The optical observation in the figure further indicates that the surface damage around the crater contains a combination of interlayer and intralayer damage. The optical observation in the figure further shows that the surface damage around the crater contains a combination of interlaminar and intralaminar damage. Our positions of interest were regions 1 to 4, delimited by the distance to the dent. These areas could be enlarged. The most severe damage can be observed in regions 1 and 2, including fiber breakage, matrix fracture, matrix crack, and interlaminar delamination, which mainly should be caused by direct compression contact with the impactor. The main damage in region 3 was matrix damage, delamination, and a little fiber fracture. Several oblique cracks in the matrix were dispersed discretely and converged at the interface to form cracks, which was delamination, and this delamination mechanism was matrix crack-induced. Region 4 was far from the dent, and the surface damage differed from the other three regions. The damage was slight, and there was no noticeable fiber or matrix damage on the surface. Only delamination was observed, and no matrix cracking occurred near the delamination. It demonstrated that the delamination in this region was not due to matrix crack induction but was more likely caused by the mismatch in stiffness between the layers.

### 3.3. Internal Damage

**Figure 8:**
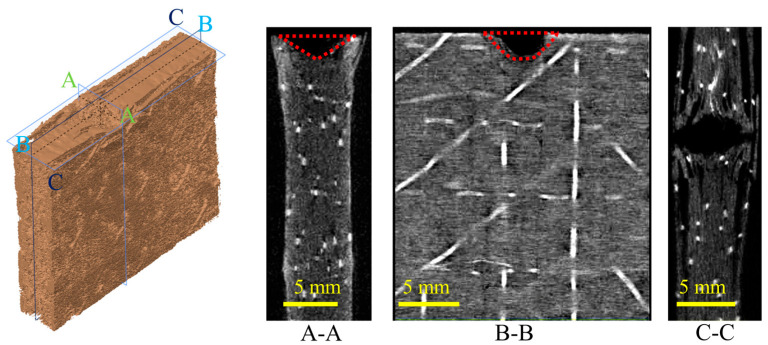
Edge-on impact morphology of composite laminate using microscopy X-ray computed tomography. section A-A represents the left view; section B-B represents the main view, and section C-C represents the top view.

The damage inside the composite laminate due to edge impact was visualized and characterized using microscopy X-ray computed tomography. A three-dimensional reconstruction was performed for a region of about 20 mm near the dented area of group SP15 laminate under 15 J. The morphology is illustrated in Figure 8. The region of the CT scan was divided into three orthogonal sections: A-A, B-B, and C-C. According to the section morphology of section A-A, it can be identified that the specimen was damaged in a region with an approximate shape of “▽”, in which there were many crushed matrix and fractured fibers near the edge that will expand and deform outward, with long cracks. As seen in sections B-B and C-C, there was a semi-elliptic damaged area in the impact area. The damaged area occurred in the highly localized area below the punch, with less damage away from the area of impact. Additionally, it was formed cumulatively by matrix transverse and longitudinal shear micro-cracks, interlaminar cracks, and fiber fractures. The dent was macroscopically expressed as tiny fragments. These small fragments slip along the fracture surface to induce interlaminar delamination, and bending fractures occur on the outer side under the influence of fragment extrusion. Matrix fracture occurred under intralayer longitudinal and transverse shear and tensile stresses, which further induced delamination and eventually permanent out-of-plane expansion, consistent with the phenomenon observed in Figure 7.

### 3.4. CAEI Damage

**Figure 9:**
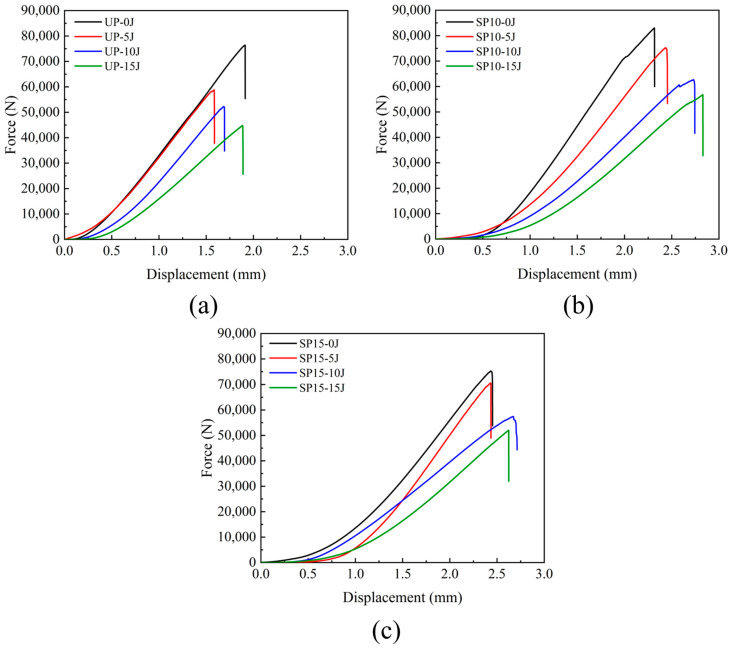
Compressive load-displacement curves for different impact energies after edge-on impact: (**a**) load-displacement curves for group UP; (**b**) load-displacement curves for group SP10; (**c**) load-displacement curves for group SP15.

The load-displacement curves under compression load were obtained by the compression testing device at the specimen loading position. The force-displacement graphs for the three groups of UP, SP10, and SP15 under different edge-on impacts are shown in Figure 9, where 0 J represents no edge impacts. As seen in Figure 9a–c, the force-displacement curves of both stitched and unstitched laminates followed the same rule. There was a maximum force for every curve, and after the point of maximum force, the compression force decreased rapidly, indicating that the specimen had been wholly damaged and could not bear any more compression force. It can be observed that when the displacement was less than a specific value, the slope of the curve increased gradually and then remained linearly increasing until the specimen was finally damaged. Figure 9b,c represent the compressive load-displacement curves for SP10 and SP15, respectively. As shown in the figures, there was little difference between the displacements of the laminates of both stitching densities during damage. However, the extreme compressive load in group SP10 was more significant than in group SP15. It may be explained by the fact that as the stitch density increased, the number of stitched resin cylinders in the laminate also increased, which improved the compressive strength of the laminate and allowed the laminate to withstand higher compressive forces.

**Figure 10:**
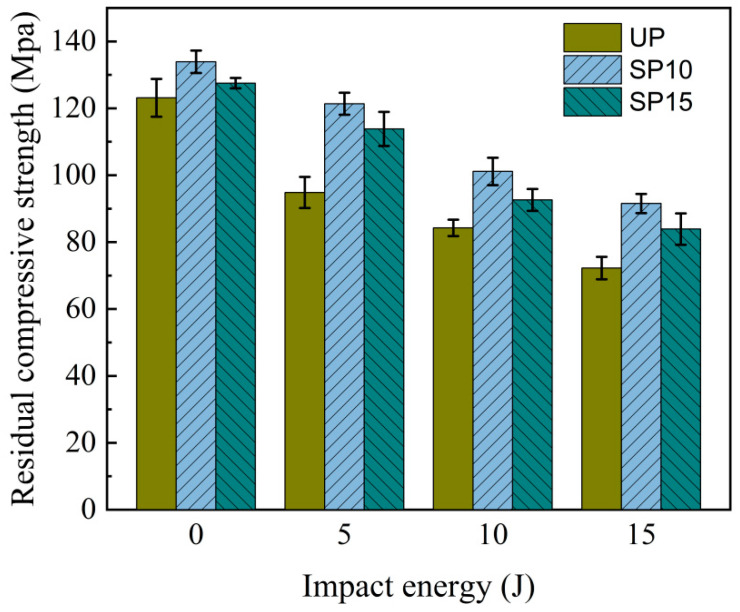
Compression residual strength under different impact energies.

The maximum residual strength was plotted in a histogram of compression residual strength-impact energy, as shown in Figure 10.

The picture shows that the relationship between residual strength and energy of impact in the three groups had the same trend under the same conditions. The residual strength decreased with the impact energy increase, but the degree of decrease was different. The residual strength of the three groups decreased by 9% to 23% when the impact energy was 5 J and by 32% to 41% when the impact energy was 15 J. This was mainly because the damage caused was more severe with the increase in energy from the impact. The residual strength of the sutured laminate decreased to a smaller extent than that of the unstitched laminate under the same energy impact. It was because the stitched resin cylinders inhibited damage extension when the laminate was impacted, which became more pronounced as the density of the stitching increased. Therefore, the residual strength performance of the sutured laminate in compression after edge impact is better than that of the unstitched laminate.

## 4. Numerical Simulations

### 4.1. Failure Criteria and Stiffness Degrading

Generally speaking, the damage to composite laminate caused by LVEI is of two types: intralaminar damage and interlaminar damage. Intralaminar damage includes fracture of fibers in tension or compression and fragmentation of the matrix in tension or compression. Interlaminar damage is damage to delamination between neighboring laminates. A progressive damage model can describe the damage process, which contains the damage initiation criterion and the damage evolution model.

#### 4.1.1. Determination Criteria for Intralaminar Damage

In this work, the Hashin failure criterion [41] is used as the initial criterion for fiber and matrix damage, and the detailed failure criterion is as follows:

1. Fiber damage

Tensile fiber damage (*σ*_11_ ≥ 0)

(1) $$F_{ft}={\left(\frac{\sigma_{11}}{X_{T}}\right)}^{2}+{\left(\frac{\sigma_{12}}{S_{12}}\right)}^{2}+{\left(\frac{\sigma_{13}}{S_{13}}\right)}^{2}\geq 1$$

Compression fiber damage (*σ*_11_ < 0)

(2) $$F_{fc}={\left(\frac{\left|\sigma_{11}\right|}{X_{C}}\right)}^{2}\geq 1$$

2. Matrix damage

Tensile matrix damage (*σ*_22_ + *σ*_33_ ≥ 0)

(3) $$F_{mt}={\left(\frac{\sigma_{22}+\sigma_{33}}{Y_{T}}\right)}^{2}+{\left(\frac{\sigma_{12}}{S_{12}}\right)}^{2}+{\left(\frac{\sigma_{13}}{S_{13}}\right)}^{2}+\left(\frac{\sigma_{23}^{2}-\sigma_{22}\sigma_{33}}{S_{23}^{2}}\right)\geq 1$$

Compression matrix damage (*σ*_22 +_ *σ*_33_ < 0)

(4) $$F_{mc}=\frac{1}{4}{\left(\frac{\sigma_{22}+\sigma_{33}}{S_{12}}\right)}^{2}+{\left(\frac{\sigma_{12}}{S_{12}}\right)}^{2}+{\left(\frac{\sigma_{13}}{S_{13}}\right)}^{2}+\left(\frac{\sigma_{23}^{2}-\sigma_{22}\sigma_{33}}{S_{23}^{2}}\right)+\left(\frac{\sigma_{22}+\sigma_{33}}{Y_{C}}\right)\left[\frac{1}{4}{\left(\frac{Y_{C}}{S_{12}}\right)}^{2}-1\right]\geq 1$$

where *F_ft_*, *F_fc_*, *F_mt_*, and *F_mc_* are the variables of the damage in the different damage modes; *σ_ij_* (*i*,*j* = 1,2,3) represents the stress in each direction; *X*_T_ and *X*_C_ represent the longitudinal tensile strength and compressive strength; *Y*_T_ and *Y*_C_ represent the transverse tensile strength and compressive strength; and *S*_12_, *S*_13_, and *S*_23_ represent the longitudinal and transverse shear strength.

#### 4.1.2. Determination Criteria for Interlaminar Damage

Interlaminar damage is a typical damage mode for composite laminates, and we introduce a zero-thickness cohesive element [42,43]. The Benzeggaagh and Kenane (B-K) criterion [44] was used to calculate the energy dissipation under mixed-mode loading, as shown in Equation (5):

(5) $$G^{C}=G_{n}^{C}+\left(G_{s}^{C}-G_{n}^{C}\right){\left(\frac{G_{S}}{G_{T}}\right)}^{\eta }$$

where *G*^C^ denotes the total critical strain energy; $G_{n}^{C}$ and $G_{s}^{C}$ are represented as the fracture energy in the direction of the normal and shear directions, respectively; *G*_S_ and *G*_T_ denote the shear dissipation energy and total dissipation energy, respectively; and η is the curtain index in the B-K criterion and is taken as 1.45 [45].

#### 4.1.3. Damage Evolution

After satisfying the damage initiation criterion, continued loading will reduce the stiffness of the laminate and the structural load-carrying capacity, so it is necessary to define reasonable stiffness degradation. The material parameters will be changed when the laminate fiber and matrix fail, and the elements in the finite element model will be distorted and lead to calculation errors when the damage is extensive. The Camanho stiffness reduction method [46] was chosen as the stiffness reduction scheme, changing the reduction factor to a control variable associated with the calculation time to prevent this situation. The details are as follows:

Tensile fracture of the fiber

(6) $$E_{11}^{d}={D^{\prime }}_{\mathrm{ft}}E_{11}$$

Compression fracture of the fiber

(7) $$E_{11}^{d}={D^{\prime }}_{\mathrm{fc}}E_{11}$$

Tensile or shear cracking of the matrix

(8) $$E_{22}^{d}={D^{\prime }}_{\mathrm{mt}}E_{22},G_{12}^{d}={D^{\prime }}_{\mathrm{mt}}G_{12},G_{23}^{d}={D^{\prime }}_{\mathrm{mt}}G_{23}$$

Compression or shear cracking of the matrix

(9) $$E_{22}^{d}={D^{\prime }}_{\mathrm{mc}}E_{22},G_{12}^{d}={D^{\prime }}_{\mathrm{mc}}G_{12},G_{23}^{d}={D^{\prime }}_{\mathrm{mc}}G_{23}$$

where ${D^{\prime }}_{\mathrm{ft}}=1-0.94{\left(t_{i}/t\right)}^{2}$; ${D^{\prime }}_{\mathrm{fc}}=1-0.86{\left(t_{i}/t\right)}^{2}$; ${D^{\prime }}_{\mathrm{mt}}=1-0.80{\left(t_{i}/t\right)}^{2}$; ${D^{\prime }}_{\mathrm{mc}}=1-0.60{\left(t_{i}/t\right)}^{2}$; $E_{11}^{d}$, $E_{22}^{d}$, $G_{12}^{d}$, and $G_{23}^{d}$ are the variables of the damage in the different damage evolution modes; *E*_11_ and *E*_22_ denote the longitudinal modulus of elasticity and the transverse modulus of elasticity; *G*_12_ and *G*_23_ denote the shear modulus; *t_i_* is the current time during the finite element model calculation; and *t* is the entire time between the initiation of tup contact with the specimen and the velocity zero of the impactor.

#### 4.1.4. Model of the Resin Cylinder with Stitching

**Figure 11:**
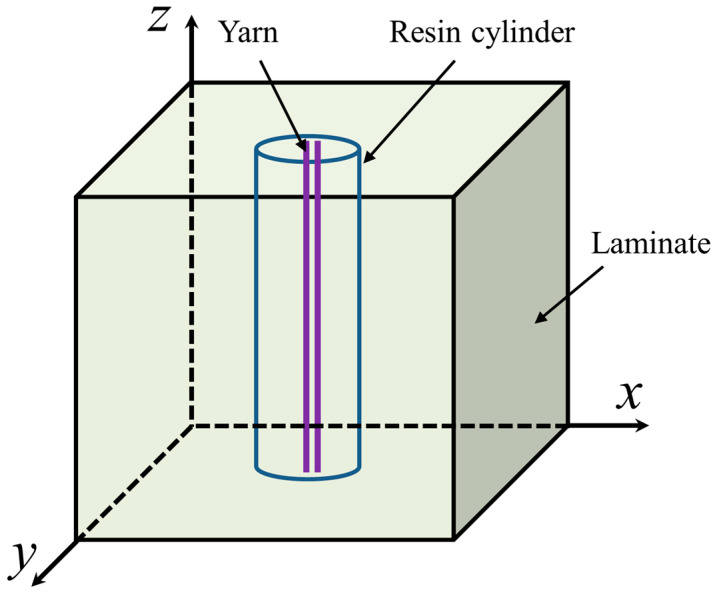
Model of the stitching resin cylinder.

Following the introduction of stitches, the resin and stitches are formed into a stitched resin cylinder after curing is completed. The stitched composite laminate can be regarded as a composite material consisting of laminates and stitched resin cylinders. The stitched resin cylinders are periodically distributed in the laminates, as seen in Figure 11. The direction *x* is the stitch pitch, the direction *y* is the stitch row pitch, and the direction *z* is through the thickness. Through homogenization theory [47], the material properties of the stitched resin cylinder are determined by the calculated volume fraction of the mixture of stitch and resin curing agent, expressed as follows:

(10) $$E=E_{S}V_{S}+E_{R}V_{R}$$

(11) $$X=X_{S}V_{S}+X_{R}V_{R}$$

(12) $$\nu =\nu_{S}V_{S}+\nu_{R}V_{R}$$

(13) $$\rho =\rho_{S}V_{S}+\rho_{R}V_{R}$$

where *E* denotes the modulus of elasticity; *X* denotes tensile strength; *v* denotes Poisson’s ratio; *ρ* denotes density; and the subscripts *S* and *R* represent the suture and resin, respectively.

The constitutive model of the stitched resin cylinder is shown in Equation (14):

(14) $$\left\{\begin{array}{c} \sigma_{11}=E\varepsilon_{11},\varepsilon_{11}\geq 0\\ \sigma_{11}=0,\varepsilon_{11}<0 \end{array}\right.$$

where *σ*_11_ is the tensile stress; *E* is the modulus of elasticity; and *ε*_11_ is the compressive stress.

Based on the maximum strain criterion, when the strain acting on the stitching resin cylinder exceeds the maximum allowable strain, it can be considered that the stitching resin cylinder fails, and the damage criterion is Equation (15).

(15) $$D_{st}={\left(\frac{\sigma }{\varepsilon_{\max}E}\right)}^{2}\geq 1$$

where *D_st_* is the damage to the stitching resin cylinder; *σ* is the stress suffered by the stitching resin cylinder; *E* is the modulus of elasticity; and *ε*_max_ is the maximum allowable strain.

### 4.2. Finite Element Model

**Figure 12:**
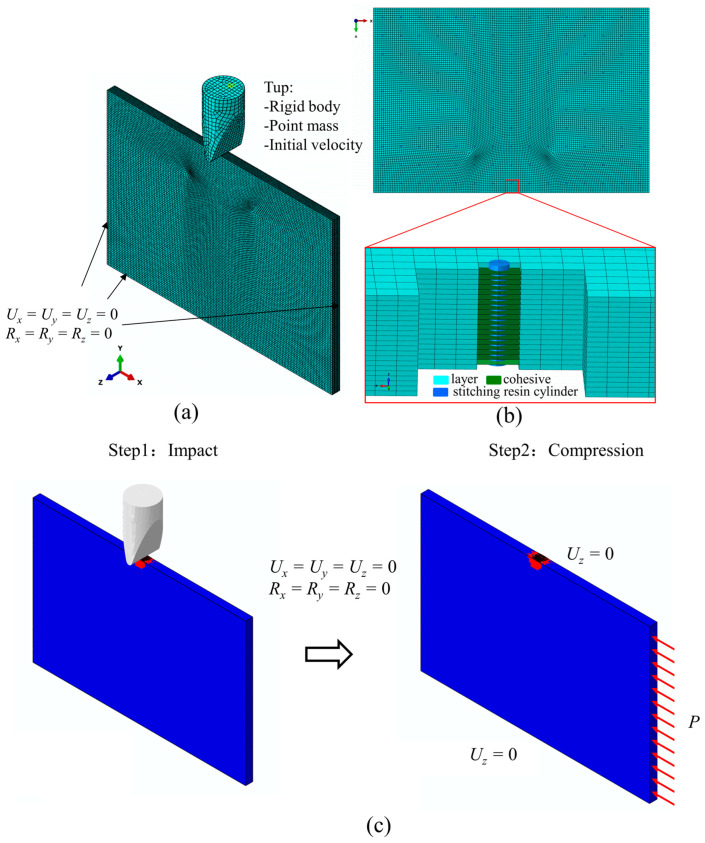
Finite element model. (**a**) Finite element model of LVEI; (**b**) stitching resin cylinder embedded into the finite element model; (**c**) steps for CAEI.

Abaqus 2016/Explicit was used for finite element modeling, and the VUMAT subroutine was written to describe the 3D Hashin failure criteria and damage evolution method. The finite element modeling process is illustrated in Figure 12. The modeling process is in two steps: the first step is to create the edge-on impact model, which is used to predict the edge impact response. The second step is to import the impact results into the CAEI model and finally predict the CAEI. The modeling of the edge impact is shown in Figure 12a, with a layup order of [−45/0/90/45/90]_2s_ and a geometric dimension defined as a length of 150 mm, a width of 100 mm, and a thickness of 6 mm. The local coordinate system defines the material layup direction, and a wedge-shaped tup is selected with a bottom chamfer radius of 2 mm. The impactor is considered a rigid body given a mass of 5.5 kg and an initial velocity calculated according to different impact energy values; the boundary conditions are kept consistent with the test. An eight-node solid element with reduced integration, C3D8R, is used for each layer, and the relaxation stiffness hourglass method is selected for mesh division to reduce the deformation during impact. An eight-node cohesive element with zero thickness, COH3D8, is inserted between two adjacent layers, and the stitching resin cylinders are simulated by T3D2 rod elements, which are embedded in the laminate whole, as shown in Figure 12b. To ensure computational accuracy, the global mesh size of the laminate is 1 × 1 × 0.3 mm, which consists of 298 200 C3D8R elements and 283 290 COH3D8 elements in total. The surface-to-surface contact algorithm was used to simulate the contact process between the impactor and the laminate. The normal contact property is set to contact hard, and the tangential contact property friction coefficient is 0.25. Once the impact prediction results are obtained, the computational results are imported into the CAEI model, as shown in Figure 12c. The fixed constraint is applied to the left side of the model. The *z*-directional constraint is applied to the top and bottom sides of the model to simulate the role of the side plate of the fixture, and the compression load P is given to the right side in the *x*-direction.

The material parameters of the stitching resin cylinder are shown in Table 3 and consist of Kevlar29 and a mixture of epoxy resin curing agents (R688/H3268), where the volume occupied by Kevlar29 is 30%. The equivalent material parameters of the stitching resin cylinder can be calculated by substituting them into Equations (6) to (9). The laminate material parameters are shown in Table 4 and include the material parameters of the unidirectional plate and interlaminar interface.

### 4.3. Analysis of Simulation Results

#### 4.3.1. Mechanical Response

**Figure 13:**
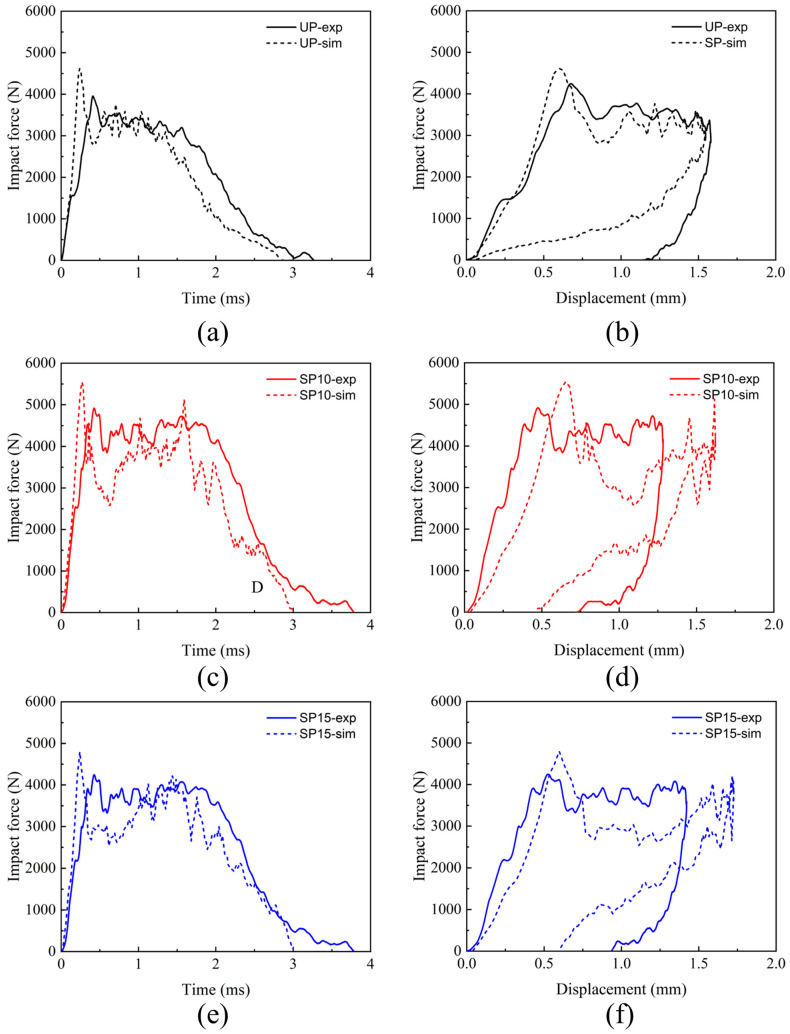
Comparison of the mechanical response of composite laminate predicted by finite elements at 5 J of edge-on impact energy with experimental results. (**a**) Force-time curves for group UP; (**b**) force-displacement curves for group UP; (**c**) force-time curves for group SP10; (**d**) force-displacement curves for group SP10; (**e**) force-time curves for group SP15; (**f**) force-displacement curves for group SP15.

The edge-on impact force-time and force-displacement curves of the three groups of specimens were compared and analyzed to verify the accuracy of the numerical simulation. We compare the simulation results at an impact energy of 5 J, as shown in Figure 13. The results of the FEM simulations better characterize the curves of the tests under edge impact loading. From Figure 13a,c,e, it can be seen that the impact force increased linearly and rapidly at the beginning of the impact process, increasing to its peak in a short time. Then the peak force decreased for a while, followed by a more extended shaking load. Finally, the force gradually unloaded as the punch rebounded. Meanwhile, it can also be found that the stitching process can improve the impact resistance threshold of the laminate, which increases with the increase in stitching density in the two densities. However, it can be seen in Figure 13b,d,f that the curves from the finite element simulation were somewhat inaccurate in the punch rebound phase compared to the experimental values. Since many fragments of fiber and matrix accumulated at the impact location during the experiment, they were constantly compressed, and these fragments rarely rebounded after unloading. Nevertheless, when the impact was unloaded during the simulation, the material would rebound, thus causing the residual displacements in the simulation to be smaller than those in the experiment. Further improvements will be needed in subsequent work to enhance the predictive capability of highly discrete feature damage.

#### 4.3.2. Progressive Damage from Edge-on Impact

**Figure 14:**
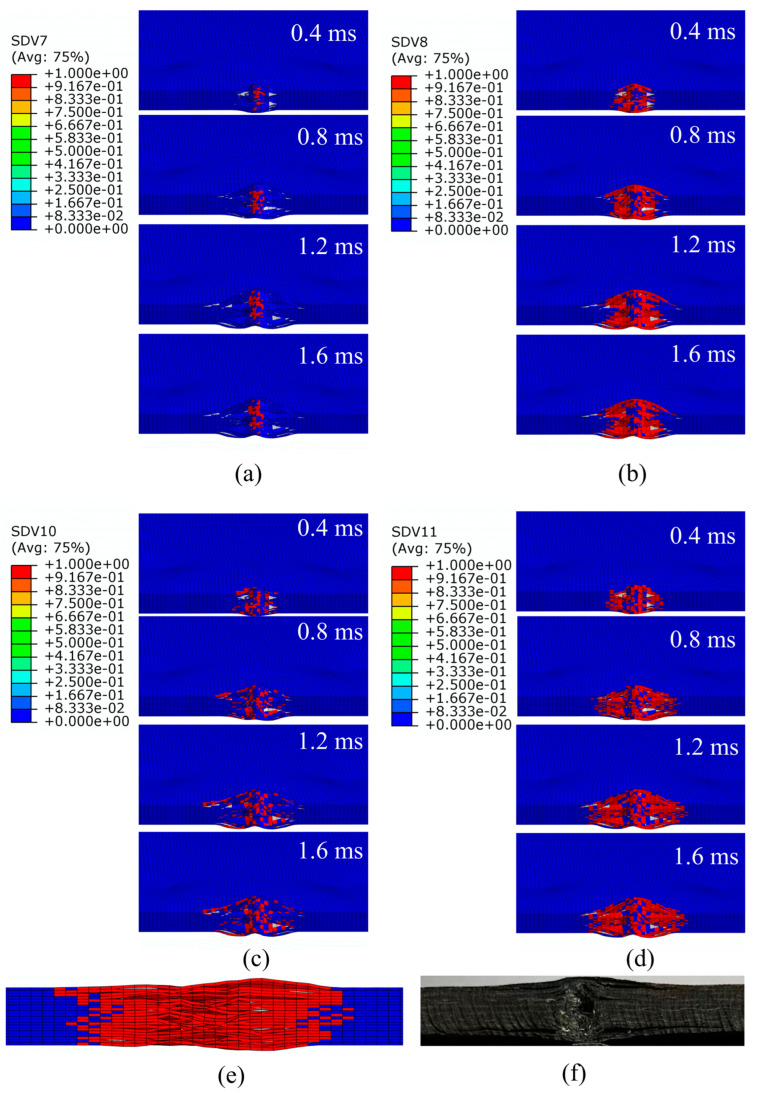
Progressive damage to composite laminate by edge impact under the energy of 15 J: (**a**) tensile failure of fiber; (**b**) compression failure of fiber; (**c**) tensile failure of matrix; (**d**) compression failure of matrix; (**e**) top view after the end of the edge-on impact in the simulation; (**f**) top view after the end of the edge-on impact in the test.

Figure 14 shows the damage process of the laminate edge-on impact, with a total time of 1.6 ms from the time the punch first touched the laminate until it started to bounce back after the punch speed had reached zero. By observing Figure 14a–d, it can be found that the compressive damage to the fibers and matrix at any moment of impact was more significant than the tensile damage. It is mainly because the bending stiffness of the laminate in the thickness direction was more significant during the impact, and the compressive stresses within the plies were in the dominant position, taking most of the impact energy. The damage process shows that the laminate developed small dents at 0.4 m, with a minor amount of fiber and matrix damage. As the impact time progressed, the area and depth of the dent also began to increase, gradually forming an elliptical crush zone that swelled and protruded outwards towards the sides of the face and extended along the longitudinal direction. The impact process ended with the final result shown in Figure 14e, from which it can be seen that there was damage to both matrix and fiber in the dent, with significant delamination near the edges. This phenomenon is consistent with Figure 14f.

#### 4.3.3. Residual Strength

A comparison between simulated and experimental results of the compressive residual strength of the laminate in compression can be seen in Table 5. As shown in the table, the residual strength of the simulation matches well with the test results, and the absolute error between the test and numerical results is less than 10%. Therefore, it can be concluded that this simulation method effectively predicts the residual strength of compression under edge-on impact.

**Figure 15:**
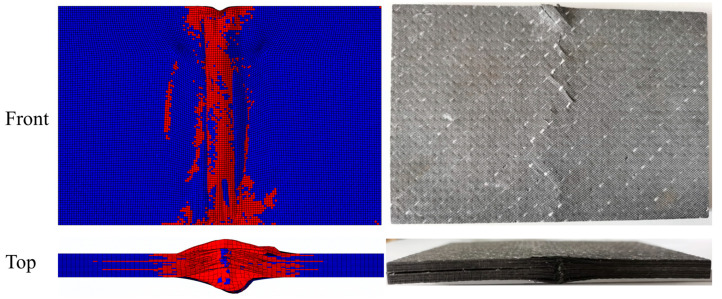
Comparison of finite element simulation results of unstitched laminate with experimental results under the energy of 15 J.

**Figure 16:**
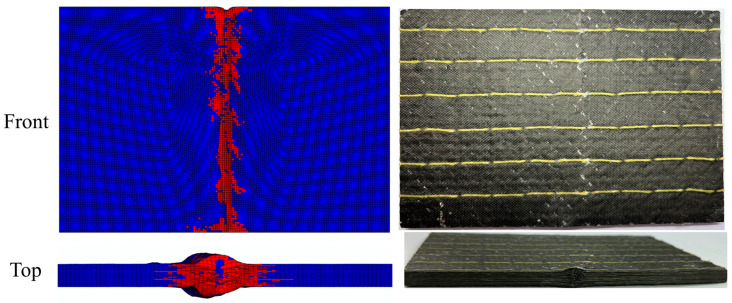
Comparison of finite element simulation results of stitched laminate with experimental results under the energy of 15 J.

Figure 15 and Figure 16 present the numerical simulation and experimental results of compression damage after impact for unstitched and stitched composite laminates, respectively. The morphology can be observed to show that their compression failure modes are obviously different. The morphology of the unstitched composite laminate after being crushed is shown in Figure 15. Truncated damage through the width direction of the specimen is seen in the center of the panel in the front view, and a long crack is found in the top view, extending along the compression direction from the impact position. As can be seen in Figure 16, the center of the laminate also shows truncated damage, which is less severe than the unstitched laminate, with cracks extending at an angle to the stitching along the stitching position. In addition, the delamination expansion is inhibited by the stitches, as can be seen in the top view. It can be concluded that the main damage to the compression under the edge-on impact of the unstitched laminate is characterized by delamination damage. While the stitched composite laminates have no evident delamination extension, the primary damage is manifested by the destruction of strength.

## 5. Conclusions

In this paper, the LVEI and CAEI properties of stitched composite laminates were investigated through experiments and numerical simulations, and the following conclusions were drawn:

(1) The edge-on impact history of laminate can be divided into a linear loading phase, a peak load abrupt drop phase, an oscillation plateau phase, and a punch rebound phase. The damage showed a high degree of localization when the peak load was reached, with noticeable dents, multiple longitudinally distributed delamination cracks, and semi-elliptical internal damage.
(2) The higher the energy of the edge impact, the more severe the internal damage to the laminate and the greater its residual displacement. The stitching process improved the edge-on impact damage tolerance of the laminate. The stitching laminate can withstand a more significant peak load at the same impact energy, and the denser the stitching, the higher the peak load. At the same time, adding stitching can inhibit the expansion of the delamination crack. The delamination crack length decreases with increasing stitch density. The depth of the dents and the degree of expansion on the sides were also smaller.
(3) A fast rise in load to the peak and then a rapid decline indicated that the specimen was damaged entirely and could not withstand the extra compression load. The higher the energy of the impact, the lower the peak loads. The stitched laminate has a higher peak load at the same energy, which increases with increasing density. The main damage to the CAEI of the unstitched laminate was characterized by delamination damage, while the primary damage to the stitched laminate was manifested by the destruction of strength. The incorporation of stitches can improve the residual strength of the laminate. At the same impact energy, the higher the stitch density, the higher the residual strength.
(4) The simulation method was developed for the LVEI and CAEI of composite laminates. Based on the Hashin failure criterion described, a cohesive zone model was used to simulate the interlaminar, combined with a modified Camanho discount degradation scheme for stiffness discounting. The mechanical response and damage predicted using the model matched well with the experimental results, which verified the accuracy of the model.

## Figures and Tables

**Table 1:** Drop height and maximum impactor velocity with three impact energies.

Impact Energy (J)	Drop Height (mm)	Maximum Impactor Velocity (m/s)
5	92.7	1.3
10	185.3	1.9
15	278.0	2.3

**Table 2:** Delamination length of specimens under three different energies.

Impact Energy (J)	Specimen Number	Delamination Length (mm)	Mean Delamination Length (mm)	Maximum Delamination Width (μm)	Maximum Mean Delamination Width (μm)
5	UP-1	19.62	21.06	310.4	332.8
	UP-2	21.04		362.1	
	UP-3	22.53		325.9	
	SP10-1	18.34	15.98	155.1	206.9
	SP10-2	14.28		206.9	
	SP10-3	15.33		258.7	
	SP15-1	14.73	17.68	258.6	258.6
	SP15-2	19.35		206.9	
	SP15-3	18.97		310.4	
10	UP-1	40.28	41.75	362.2	431.1
	UP-2	42.57		413.8	
	UP-3	42.39		517.2	
	SP10-1	23.22	26.72	235.1	327.9
	SP10-2	30.42		362.1	
	SP10-3	26.53		386.6	
	SP15-1	32.29	33.21	465.5	399.6
	SP15-2	31.28		416.1	
	SP15-3	36.04		317.3	
15	UP-1	68.38	69.73	1034.5	948.3
	UP-2	70.25		827.6	
	UP-3	70.56		982.7	
	SP10-1	45.46	45.97	724.1	655.1
	SP10-2	47.69		568.9	
	SP10-3	44.77		672.4	
	SP15-1	50.72	50.32	775.9	789.1
	SP15-2	51.56		812.1	
	SP15-3	48.68		779.1	

**Table 3:** Material parameters of the stitching resin cylinder.

Material Parameters	Kevlar29	R688-H3268	Equivalent Tricot Resin Cylinder
Yong’s modulus (GPa)	70.50	3.90	23.88
Strength (GPa)	2.92	0.08	0.93
Poisson’s ratio	0.36	0.30	0.32
Density (kg·m^−3^)	1440	1065	1178

**Table 4:** Material parameters of the CF1200-6300/R668 unidirectional plate and interlaminar interface.

Unidirectional Lamina	
Density (kg·m^−3^)	1760
Yong’s modulus (GPa)	*E*_11_ = 123; *E*_22_ = *E*_33_ = 10.1; *G*_12_ = *G*_13_ = 4.6; *G*_23_ = 3.082
Poisson’s ratio	*μ*_12_ = *μ*_13_ = 0.28; *μ*_23_ = 0.21;
Strength (GPa)	*X*_T_ = 2260; *X*_C_ = 1370; *Y*_T_ = 51; *Y*_C_ = 130; *S*_12_ = 68; *S*_13_ = S_23_ = 40
**Interface**	
Yong’s modulus (GPa)	*E* = 9.5; *G* = 8.1
Strength (GPa)	*N* = 50; *S* = 110
Fracture energy (N·mm^−1^)	$G_{n}^{C}=0.27;G_{s}^{C}=0.49$

**Table 5:** Comparison of residual strength between simulations and tests.

Edge-on Impact Energy (J)	Group	Value of Experiment (MPa)	Average Value of Experiment (MPa)	Average Value of Simulation (MPa)	Error (%)
5	UP-1	95.16	94.84	100.3	5.76
	UP-2	93.75			
	UP-3	95.62			
	SP10-1	120.79	121.35	109.37	−9.87
	SP10-2	120.92			
	SP10-3	122.34			
	SP15-1	111.42	113.85	102.89	−9.36
	SP15-2	115.89			
	SP15-3	114.25			
10	UP-1	85.79	84.24	80.14	−4.87
	UP-2	87.48			
	UP-3	82.45			
	SP10-1	101.14	101.14	99.2	−1.92
	SP10-2	103.45			
	SP10-3	98.83			
	SP15-1	93.81	92.61	88.81	−4.11
	SP15-2	93.65			
	SP15-3	90.37			
15	UP-1	70.65	72.23	74.23	2.76
	UP-2	74.63			
	UP-3	71.42			
	SP10-1	90.85	91.55	94.68	3.42
	SP10-2	95.25			
	SP10-3	88.56			
	SP15-1	86.65	83.89	82.21	−2.01
	SP15-2	81.43			
	SP15-3	83.59			

## Data Availability

Not applicable.
